# Two unusual cases of successful treatment of hypermucoviscous *Klebsiella pneumoniae* invasive syndrome

**DOI:** 10.1186/s12879-016-2011-3

**Published:** 2016-11-16

**Authors:** Hiroki Namikawa, Koichi Yamada, Hiroki Fujimoto, Ken-Ichi Oinuma, Yoshihiro Tochino, Yasuhiko Takemoto, Yukihiro Kaneko, Taichi Shuto, Hiroshi Kakeya

**Affiliations:** 1 Department of Infection Control Science, Osaka City University, Graduate School of Medicine, 1-4-3, Asahi-machi, Abeno-ku, Osaka 545-8585 Japan; 2 Department of Medical Education and General Practice, Osaka City University, Graduate School of Medicine, 1-4-3, Asahi-machi, Abeno-ku, Osaka 545-8585 Japan; 3 Department of Bacteriology, Osaka City University, Graduate School of Medicine, 1-4-3, Asahi-machi, Abeno-ku, Osaka 545-8585 Japan

**Keywords:** Hypermucoviscous *Klebsiella pneumoniae*, Abscess, Infected aneurysm, Endophthalmitis, Prostate, *magA*, *rmpA*, String test

## Abstract

**Background:**

A few Japanese cases of hypermucoviscous *Klebsiella pneumoniae* (*K. pneumoniae*) invasive syndrome have recently been reported. Although extrahepatic complications from bacteremic dissemination have been observed, infected aneurysms are rare. Furthermore, the primary source of infection is generally a liver abscess, and is rarely the prostate. Therefore, we report two atypical cases of hypermucoviscous *K. pneumoniae* invasive syndrome.

**Case presentation:**

The first case was an 81-year-old Japanese man with no significant medical history, who was referred to our hospital for vision loss in his right eye. Contrast-enhanced whole-body computed tomography revealed abscesses in the liver and the prostate, and an infected left internal iliac artery aneurysm. Contrast-enhanced head magnetic resonance imaging revealed brain abscesses. Cultures of the liver abscess specimen and aqueous humor revealed *K. pneumoniae* with the hypermucoviscosity phenotype, which carried the *magA* gene (mucoviscosity-associated gene A) and the *rmpA* gene (regulator of mucoid phenotype A). We performed enucleation of the right eyeball, percutaneous transhepatic drainage, coil embolization of the aneurysm, and administered a 6-week course of antibiotic treatment. The second case was a 69-year-old Japanese man with diabetes mellitus, who was referred to our hospital with fever, pollakiuria, and pain on urination. Contrast-enhanced whole-body computed tomography revealed lung and prostate abscesses, but no liver abscesses. Contrast-enhanced head magnetic resonance imaging revealed brain abscesses. The sputum, urine, prostate abscess specimen, and aqueous humor cultures revealed *K. pneumoniae* with the hypermucoviscosity phenotype, which carried *magA* and *rmpA*. We performed enucleation of the left eyeball, percutaneous drainage of the prostate abscess, and administered a 5-week course of antibiotic treatment.

**Conclusions:**

Hypermucoviscous *K. pneumoniae* can cause infected aneurysms, and the prostate can be the primary site of infection. We suggest that a diagnosis of hvKP invasive syndrome should be considered in all patients who present with *K. pneumoniae* infection and multiple organ abscesses.

## Background

*Klebsiella pneumoniae* (*K. pneumoniae*) is a common pathogen in community-acquired and nosocomial infections [1]. However, a new type of *K. pneumoniae* invasive syndrome has been identified in southeast Asia during the last 2 decades [2]. In the 1980s, a community-dwelling Taiwanese patient presented with a primary liver abscess that involved *K. pneumoniae* [3]. In Japan, a few recent studies have reported pyogenic infections that were caused by *K. pneumoniae* [4, 5]. Extrahepatic complications have also been observed because of bacteremic dissemination, such as endophthalmitis, meningitis, and other diseases. However, infected aneurysms caused by hypermucoviscous *K. pneumoniae* (hvKP) are relatively rare. A liver abscess may occur after leakage of *K. pneumoniae* from the intestinal epithelium and bacterial translocation into the liver via the portal circulation [6]. However, hvKP bacteremia involving the prostate via the ascending route is extremely rare. In this context, hypermucoviscous strains are identified using the appearance of colonies that are grown on an agar plate. Furthermore, several studies have indicated that hypermucoviscosity is associated with the mucoviscosity-associated gene A (*magA*) and the regulator of mucoid phenotype A (*rmpA*) genes [7, 8].

We report two rare cases of hvKP invasive syndrome that involved strains that were positive for *magA* and *rmpA*. The first case involved an 81-year-old man with hvKP invasive syndrome who presented with brain, liver, and prostate abscesses; bacterial endophthalmitis; and an infected aneurysm. The second case involved a 69-year-old man with hvKP invasive syndrome who presented with brain, lung, and prostate abscesses; bacterial endophthalmitis; but no liver abscesses.

## Case presentation

### Case 1

**Fig. 1:**
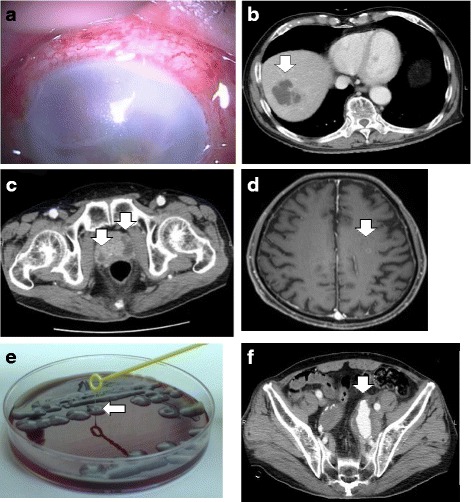
Findings from the first case. **a** Findings of remarkable edema and opacity in the cornea. **b** Contrast-enhanced whole-body computed tomography (CT) reveals a liver abscess (*white arrow*). **c** Contrast-enhanced whole-body CT reveals prostate abscesses (*white arrows*). **d** Contrast-enhanced head magnetic resonance imaging reveals a brain abscess (*white arrow*). **e** The first case provided colonies on the agar plate that stretched for >5 mm using the inoculation loop (*white arrows*), which indicates a positive string test result. **f** Contrast-enhanced whole-body CT reveals the enlargement of an existing left internal iliac artery aneurysm (*white arrow*)

An 81-year-old man was referred to our hospital with a 10-day history of vision loss in his right eye. He also exhibited ophthalmalgia and general malaise. There was no relevant medical history, no history of alcoholism, and no use of prescription medication. He had no recent travel history, including within Southeast Asia, and no history of contact with animals. A physical examination revealed that his vital signs were stable, although he had swelling of the right eyelid and corneal edema in the right eye (Fig. 1a). Blood tests revealed a white blood cell count of 11,700/mm^3^, an albumin level of 2.5 g/dL, and a C-reactive protein level of 6.46 mg/dL. Contrast-enhanced whole-body computed tomography (CT) revealed liver and prostate abscesses (Fig. 1b and c), and contrast-enhanced head magnetic resonance imaging (MRI) revealed brain abscesses (Fig. 1d). However, a urine culture and two sets of blood cultures yielded negative results. *K. pneumoniae* was cultured from both the liver abscess specimen and the aqueous humor. Isolate identification was confirmed using the MicroScan WalkAway-96 SI system (Beckman Coulter Inc., Brea, CA, USA). The minimum inhibitory concentrations were also determined using the MicroScan WalkAway-96 SI. The *K. pneumoniae* was susceptible to all tested antimicrobials, with the exception of ampicillin. The agar plate colonies appeared hypermucoviscous and the string test yielded positive results (Fig. 1e). Therefore, we diagnosed the patient with hvKP invasive syndrome; brain, liver, and prostate abscesses; and bacterial endophthalmitis.

We performed enucleation of the right eyeball and percutaneous transhepatic drainage, and administered cefotaxime (6 g/day). Although the antibiotic treatment was continued for 9 days, it had little effect on the fever and inflammatory markers. Therefore, the antibiotic treatment was switched to levofloxacin (500 mg/day), and subsequently to meropenem (6 g/day). This reduced the fever and improved the inflammatory marker levels. However, the patient subsequently reported left lower quadrant abdominal pain. Contrast-enhanced abdominal CT revealed enlargement of an existing left internal iliac artery aneurysm (Fig. 1f), and we diagnosed the patient with infected hvKP aneurysm and impending rupture. Based on the patient’s age and risk factors, we successfully performed coil embolization instead of surgery. He subsequently developed a rash over his body, which we attributed to the meropenem. Thus, the antibiotic regimen was switched to cefotaxime (6 g/day) with levofloxacin (500 mg/day). After a 6-week course of treatment, the patient’s inflammatory marker levels returned to normal, and we observed reductions in the sizes of the brain, liver, and prostate abscesses. The antibiotic therapy is ongoing, and there has been no symptom recurrence.

### Case 2

**Fig. 2:**
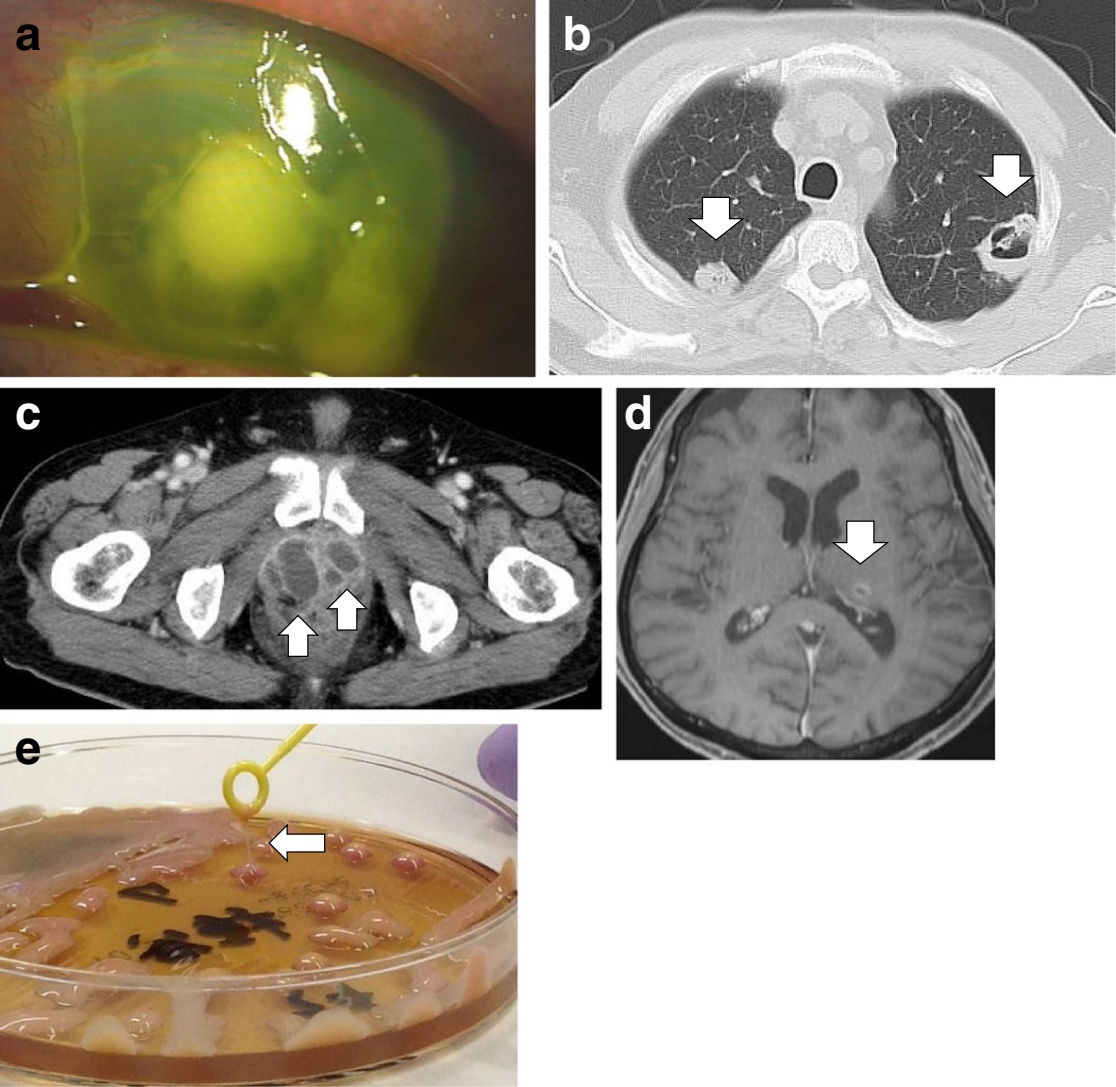
Findings from the second case. **a** Findings of remarkable edema and opacity in the cornea. **b** Contrast-enhanced whole-body computed tomography (CT) reveals lung abscesses (*white arrows*). **c** Contrast-enhanced whole-body CT reveals prostate abscesses (*white arrows*). **d** Contrast-enhanced head magnetic resonance imaging reveals a brain abscess (*white arrow*). **e** The second case provided colonies on the agar plate that stretched for >5 mm using the inoculation loop (*white arrows*), which indicates a positive string test result

A 69-year-old man was referred to our hospital with a 10-day history of fever, pollakiuria, and pain on urination. He also exhibited vision loss in his left eye and a wet cough. The patient had a history of diabetes mellitus (DM) and hypertension, although there was no history of alcoholism. He had no recent travel history, including within Southeast Asia, and no history of contact with animals. A physical examination revealed that his blood pressure was 149/87 mmHg, his pulse was 122 beats per minute, his body temperature was 38.7 °C, and his oxygen saturation was 93% in room air. We detected no abnormal findings during auscultation, and there was no costovertebral angle tenderness, although we detected corneal opacity in his left eye (Fig. 2a). Blood tests revealed a white blood cell count of 23,600/mm^3^, an albumin level of 2.6 g/dL, a C-reactive protein level of 26.22 mg/dL, and a hemoglobin A1c level of 8.1%. Contrast-enhanced whole-body CT revealed lung and prostate abscesses (Fig. 2b and c), but no liver abscesses. Contrast-enhanced head MRI revealed a brain abscess (Fig. 2d). *K. pneumoniae* was cultured from the sputum, urine, prostate abscess specimen, and aqueous humor. Isolate identification was confirmed using the MicroScan WalkAway-96 SI system (Beckman Coulter Inc., Brea, CA, USA). The minimum inhibitory concentrations were also determined using the MicroScan WalkAway-96 SI. The *K. pneumoniae* was susceptible to all tested antimicrobials, with the exception of ampicillin. The agar plate colonies appeared hypermucoviscous and the string test yielded positive results (Fig. 2e). Therefore, we diagnosed the patient with hvKP invasive syndrome; brain, lung, and prostate abscesses; and bacterial endophthalmitis.

We performed enucleation of the left eyeball and percutaneous drainage of the prostate abscess. We initially administered tazobactam/piperacillin (18 g/day), which was switched to intravenous levofloxacin (500 mg/day) based on drug migration to the prostate. The antibiotic regimen was continued for 7 days, although the patient’s fever and inflammatory markers exhibited little improvement. Thus, we changed the antibiotic treatment to meropenem (6 g/day), which improved the fever and inflammatory markers, and we subsequently switched the patient to oral levofloxacin (500 mg/day) for maintenance therapy. After a 5-week course of treatment, we did not detect any markers of inflammation, and the brain, lung, and prostate abscesses had decreased in size. Therefore, the antibiotic treatment was discontinued and there has been no symptom recurrence.

### Genetic analysis

**Fig. 3:**
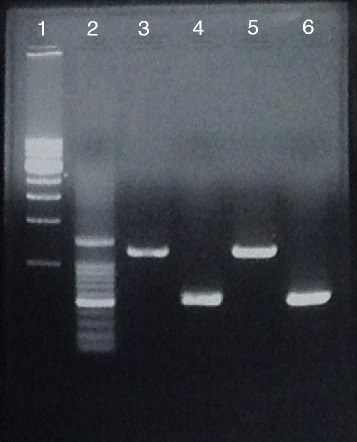
Agarose gel electrophoresis. Lane 1: 1–10 kbp DNA ladder; Lane 2: 100–1,500 bp DNA ladder; Lanes 3 and 4: the PCR product reveals *magA* and *rmpA* in the first patient; Lanes 5 and 6: the PCR product reveals *magA* and *rmpA* in the second patient

Polymerase chain reaction (PCR) assays of the isolates from both cases were performed as previously described [9], and revealed the presence of *magA* and *rmpA* (Fig. 3). Multilocus sequence typing (MLST) was performed according to the methods of Diancourt et al. [10]. The MLST revealed sequence type 23 in both cases.

## Discussion

*K. pneumoniae* is a common human pathogen that causes pneumonia and urinary tract infections (UTIs). In recent years, an increased incidence of hvKP syndrome has been reported in Taiwan [11], and this syndrome is associated with bacteremia, primary liver abscess, and metastatic infections [6]. Extrahepatic complications have also been observed because of bacteremic dissemination, such as endophthalmitis, meningitis, and other diseases. However, to the best of our knowledge, there are few reports of infected aneurysms that were caused by hvKP [12, 13]. In Case 1 from the present report, the patient reported new-onset left lower quadrant pain, and contrast-enhanced abdominal CT revealed enlargement of an existing left internal iliac artery aneurysm. Although we did not have positive culture results from the aneurysm tissue, the diagnosis of hvKP infection was made based on our clinical and diagnostic findings (new-onset left lower quadrant pain and enlargement of the existing left internal iliac artery aneurysm despite appropriate antibiotic treatment). A previous report has indicated that DM and an age of <65 years were independent predictors of metastatic ocular or central nervous system complications of pyogenic liver abscesses [14]. Another report has identified a history of alcoholism and DM as significant risk factors for the development of metastatic complications from pyogenic liver abscesses [15]. However, the patient in Case 1 had no known risk factors, as he was 81 years old and had no history of DM or alcoholism. In addition, a previous report has indicated that glycemic control in patients with DM played an important role in the clinical characteristics of KP liver abscesses, especially in metastatic complications from KP liver abscess [16]. The patient in Case 2 took vildagliptin (100 mg/day) and his hemoglobin A1c was 8.1%; therefore glycemic control was relatively poor. It is suggested that the poor glycemic control played an essential role in dissenminated KP infection.

The mechanisms for classic *K. pneumoniae* entry into extraintestinal sites include ascension from the perineum into the bladder, disruption of the bowel enabling entry of gastrointestinal tract colonizers into the peritoneal cavity, and aspiration of oropharyngeal colonizers into the respiratory tract [17]. However, the mechanism for hvKP remains unclear, and it is currently speculated that leakage of *K. pneumoniae* from a patient's intestinal mucosa, and bacterial translocation into the liver via the portal circulation, results in liver abscesses and bacteremic dissemination [6]. In Case 2, the patient presented with fever, pollakiuria, and pain on urination, which was indicative of a UTI. Furthermore, contrast-enhanced whole-body CT revealed prostate abscesses, but no liver abscesses. These findings suggested a primary prostate lesion, although the ascending route of infection via the prostate is an extremely rare cause of hvKP bacteremia. Moreover, this patient presented with disseminated lesions secondary to the prostate infection, which is extremely rare. Unlike liver abscesses, the K1 serotype of *K. pneumoniae* is considered closely related to hematogenous metastasis, and is rarely detected in the urinary tract [18]. In addition, clinicians may reach a rapid diagnosis of a UTI based on its typical symptoms, such as micturition pain, constant urge to urinate, and back pain. Therefore, we speculate that there are few reports of UTIs that were caused by hvKP, as antibiotic treatment would typically be started before the development of disseminated abscesses.

MLST has been used as a nucleotide sequence-based method for characterizing microorganisms [19]. This approach is particularly useful for the typing of microbial pathogens, in order to identify clones with noticeably different virulence characteristics [20]. Invasive syndrome caused by highly virulent strains with sequence type 23 has become increasingly common in Southeast Asia [21], and the MLST revealed sequence type 23 in both of the present cases.

The hypermucoviscous phenotype can easily be confirmed using the string test [6]. In this context, a positive result is defined as bacterial colonies on an agar plate stretching for >5 mm using the inoculation. Thus, both of the present cases exhibited positive results for the hypermucoviscous phenotype, based on the string test results. Furthermore, the *magA* and *rmpA* genes are related to the expression of the hypermucoviscous phenotype [7]. In this context, the *magA* gene encodes an outer membrane protein that is essential for the formation of a protective exopolysaccharide web, which is related to the bacterial virulence and mucoviscosity of the K1 serotype [22]. The enzyme encoded by *magA* gene functions as a polymerase involved in capsule synthesis, and this function is restricted to the capsular gene cluster of K1 serotype only [6]. In contrast, the *rmpA* gene is a plasmid-borne regulator of extracellular polysaccharide synthesis [23]. Moreover, the *rmpA* gene is strongly associated with abscess formation [7].

## Conclusions

In conclusion, we report two rare cases of hvKP invasive syndrome that involved strains that were positive for *magA* and *rmpA*. Therefore, we suggest that a diagnosis of hvKP invasive syndrome should be considered in all patients who present with *K. pneumoniae* infection and multiple organ abscesses.

## Abbreviations

CT: computed tomography
DM: diabetes mellitus
hvKP: hypermucoviscous *K. pneumoniae*
*K. pneumoniae*: *Klebsiella pneumoniae*
*magA*: mucoviscosity-associated gene A
MLST: multilocus sequence typing
MRI: magnetic resonance imaging
PCR: polymerase chain reaction
*rmpA*: the regulator of mucoid phenotype A
UTI: urinary tract infection
